# On Modeling and Optimization for Separation, Concentration, and Purification of Saponins and Phenolic Compounds from Quinoa Hulls by Nanofiltration

**DOI:** 10.3390/membranes16070228

**Published:** 2026-07-01

**Authors:** Ana I. García López, Javier M. Ochando Pulido, Mercedes Fernández Serrano, Germán Luzón González, Josefa Núñez-Olea, Natalia Chaves

**Affiliations:** Department Chemical Engineering, Granada University, Fuente Nueva s/n, 18071 Granada, Spainmferse@ugr.es (M.F.S.); german@ugr.es (G.L.G.);

**Keywords:** circular economy, saponins, polyphenols, valorization, extraction, nanofiltration

## Abstract

It is essential for quinoa’s rapid expansion in the global market to comply with the circular economy to become a green agro-food industry. For this purpose, in this work, bioactive added-value compounds, specifically saponins and phenolic antioxidants, were extracted and purified from quinoa by-products (QbP), namely hulls, using *green* solvent extraction (60 wt% ethanol-water) and nanofiltration (NF). So far, research published on the implementation of NF in the treatment of QbP, or modelization and optimization of the membrane performance focusing on fouling minimization and control, is scarce. Centrifugation and microfiltration were conducted as separation-purification pretreatments before NF. A three-level factorial design was successfully applied to optimize NF membrane operation in terms of saponins and phenolic compound recovery, as well as permeate flux, comprising operating pressure and tangential velocity as key input factors. Membrane fouling, critical for stable process operation scale-up, required intensive multifactorial analysis. Optimization at 4 bar and 15 m/s permitted the recovery of up to 84.5% saponins and 84.3% phenolic compounds in the permeate stream. Moreover, NF dynamic performance modeling and optimization ensured fouling build-up minimization and maximization of membrane productivity almost ten-fold, up to a stable value as high as 175.4 L/hm^2^, ensuring full recovery of the membrane performance after each operating cycle, key for the technical–economic viability of the proposed process to obtain standardized purified extract products.

## 1. Introduction

The shift toward sustainable chemistry has placed natural surfactants at the forefront of industrial innovation. As the detergent, cosmetic, and food sectors move away from synthetic additives—which are increasingly scrutinized for their low biodegradability and cumulative toxicity in groundwater and various ecosystems [1,2,3,4]—the revalorization of agricultural by-products has become a strategic necessity.

Quinoa (*Chenopodium quinoa*) is globally celebrated for its nutritional density, boasting a protein content of 16–23% and a rich profile of omega fatty acids [5,6,7]. However, its industrial processing generates a significant volume of vegetable residue: the hulls. These hulls are traditionally removed to eliminate bitterness but are, in fact, a concentrated source of amphiphilic glycosides, specifically saponins [8,9,10,11], as well as a diverse array of polyphenols [12,13,14,15].

Saponins are natural biosurfactants composed of a lipophilic aglycone and a hydrophilic glycone [16,17]. This structure allows them to reduce surface tension and stabilize emulsions, making them ideal replacements for synthetic foaming and wetting agents. Beyond their functional utility, these compounds offer a suite of bioactivities—including antimicrobial, anti-inflammatory, and antioxidant properties [11,12,13]—that provide a “dual-action” benefit in pharmaceutical and cosmetic formulations.

While the industry is transitioning toward “green” extraction methods like ultrasound and microwave-assisted extraction [8,10,18,19,20,21,22,23] to avoid the deleterious safety and environmental concerns of organic solvents, the real challenge lies in purification. Traditional methods, such as column chromatography or solvent partitioning, are often energy-intensive and difficult to scale, frequently resulting in high costs that hinder industrial adoption [24,25,26,27,28].

Saponins found in plants, like soy and quinoa, typically possess a molecular weight range of 500 to over 2000 Daltons, whereas low molecular weight (LMW) phenolic compounds typically range from roughly 0.1 to 1 kDa [27,28,29,30]. To bridge the gap between laboratory-scale extraction and industrial application, membrane technology emerges as the most critical tool for the circular economy [29]. Unlike conventional thermal or chemical separation (such as chemical oxidation or coagulation [30,31,32,33,34]), membrane processes [28,29]—ranging from Microfiltration (MF) and Ultrafiltration (UF) to Nanofiltration (NF) and Reverse Osmosis (RO)—offer a sophisticated, physical separation mechanism [35,36,37,38,39].

The importance of membrane technology in this context is paramount for several reasons: its selectivity and purity; the stability of bioactive compounds given that membrane separation occurs at standard conditions without phase changes; the delicate antioxidant properties of polyphenols can be preserved, which prevents the cell-level damage or oxidation that can occur with high-temperature processing [38,39]; its efficiency and sustainability since these systems operate with relatively low energy consumption and require no additives, aligning perfectly with the need to replace synthetic surfactants with biodegradable alternatives; and its scalability, owing to the modular nature of membrane units allows for straightforward scale-up and integration into hybrid processes [29], making it feasible to treat large volumes of quinoa hull waste and transform it into a continuous stream of high-value compounds. While the purification of saponins by membrane technologies remains underexplored, ultrafiltration, nanofiltration, and diafiltration might be uniquely suited to separating saponins from seeds and other residues [29].

The present research paper focuses on the revalorization of quinoa by-products, in particular quinoa hulls, for efficient extraction, recovery, and concentration of saponins and phenolic compounds. Maceration of quinoa hulls was performed, followed by separation, concentration, and purification of the extracted compounds by modern NF membrane technology. The main processes’ parameters were studied and optimized by using statistical tools and modeled by response surface methodologies. The whole proposed environmentally friendly process was optimized in terms of yield to permit cost reductions and enhance the circular economy of this growing agro-industry.

## 2. Materials and Methods

### 2.1. Extraction of Bioactive Compounds from Quinoa Hulls

**Figure 1:**
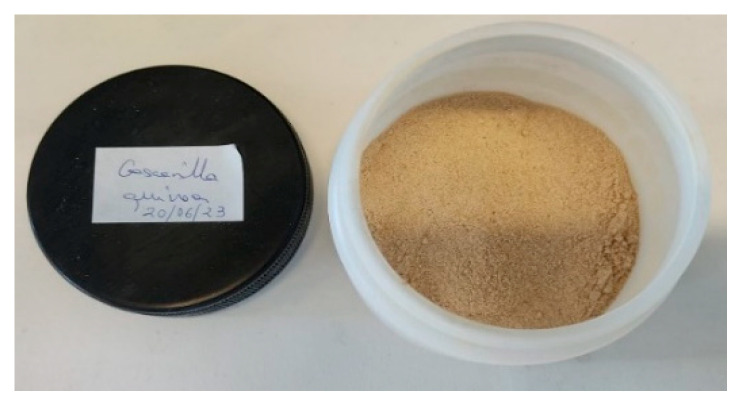
Milled quinoa hull samples used in this study.

Quinoa hull samples of the Verragosa variety provided by Iberoquinoa S.L. (Antequera, Málaga, Spain) were used for this research study. Samples were milled (1 ± 0.2 mm mean particle size) and preserved in a dry, dark environment (see Figure 1).

**Figure 2:**
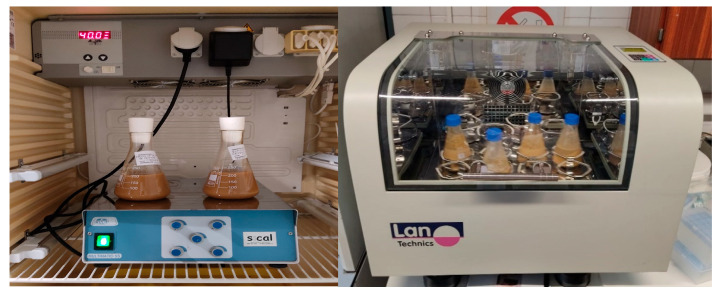
Extraction of bioactive compounds from milled quinoa hulls (magnetic agitation vs. orbital shaking).

Thereafter, the extraction of bioactive compounds from milled quinoa hulls was performed using the maceration procedure between 24 and 48 h with a 60 wt% ethanol-water mixture. Extraction was carried out using a 1:10 solid–liquid (extractant solvent) ratio, whereas the temperature was controlled at 40 °C and the stirring speed was set at 200 rpm to ensure complete mixing in Pyrex Erlenmeyer flasks (250 mL). Two different stirring systems (Figure 2) were used for comparison purposes: orbital shaking in an incubator (Lan. Technics MOD.200, Labolan, Spain) vs. magnetic stirring in an oven (Selecta, Holdcold S, Barcelona, Spain), in order to evaluate less shear-stressing vs. more scalable stirring systems.

After extraction, solid–liquid separation was performed using centrifugation (Hettich zentrifugen universal 320) at 9000 rpm for 5 min. Subsequently, the obtained extract was filtered under a vacuum with a glass microfiber filter (diameter Ø = 47 mm; mean pore size = 1.6 μm). Each filter piece was previously rinsed with distilled water and dried in an oven at 100 °C for 1 h.

### 2.2. Quantification of Saponin Compounds

Quantification of saponin compounds was performed by afrosymetric and spectrophotometric methods. Saponin standards (Saponin, CAS-No. 8047-15-2 from Sigma-Aldrich, Darmstadt, Germany) were used to prepare the calibration curves.

Spectrophotometric analyses were carried out using Lieberman Burchard colorimetric reagent. Readings were carried out at 416 nm wavelength in a double-beam Spectrophotometer (UV 6300 PC, VWR). Lieberman Burchard’s colorimetric reagent was prepared in a 5:1 ratio with concentrated sulfuric acid and anhydrous acetic acid, respectively. As this reagent is not completely stable over time, it was prepared for each quantification test, with both cooling and mixing being crucial factors for the effective preparation of the colorimetric reagent [40].

Moreover, the afrosymetric or foam method is based on the surfactant properties of saponins, which, when dissolved in water and after agitation, generate a stable foam whose height correlates with the saponin [41]. This process was carried out in samples diluted in water at 1:10 and 1:50 ratios, as well as undiluted samples: 1 mL of each sample was taken and added to 4 mL of distilled water. Measurements were performed in triplicate. Each tube was capped and shaken vigorously for 30 s, then left to react for 30 min. The tubes were shaken again for 20 s, left for another 30 min, and shaken again for 30 s with a final strong agitation. After standing for 5 min, the foam height was measured using a 0.1 mm precision rack.

### 2.3. Quantification of Total Phenolic Compounds

For the determination of total phenolic compounds (TPhs), the Folin–Ciocalteu method was carried out. This method is based on the reaction, in a basic medium, of phenolic compounds with sodium molybdate and sodium tungstate, components of Folin–Ciocalteu’s reagent. This reaction produces a blue coloration in the samples due to electron transfer that reduces phosphomolybdic-phosphotungstic complexes into tungsten oxides (W_8_O_23_) and molybdenum oxides (Mo_8_O_23_). The intensity of blue coloration is proportional to the number of hydroxyl groups and was quantified spectrophotometrically at a wavelength of 765 nm [42].

To determine the total phenolic content, 100 μL of the sample, standard solution, or distilled water (for the blank) was pipetted and diluted with 8 mL of distilled water, followed by the addition of 500 μL of the Folin–Ciocalteu reagent. The mixture was homogenized using a *Stuart SA7* vortex mixer and allowed to stand for 5 min before adding 1.5 mL of sodium carbonate solution. After a second vortexing step, the mixture was incubated in an oven at 40 °C for 30 min. Finally, the samples were allowed to cool for 10 min, and the absorbance was measured. To obtain the calibration curve for TPhs quantification, gallic acid of 99% purity was used as a standard.

### 2.4. Bench-Scale Membrane Plant, Characteristics of Selected Membrane, and Operational Setup

**Figure 3:**
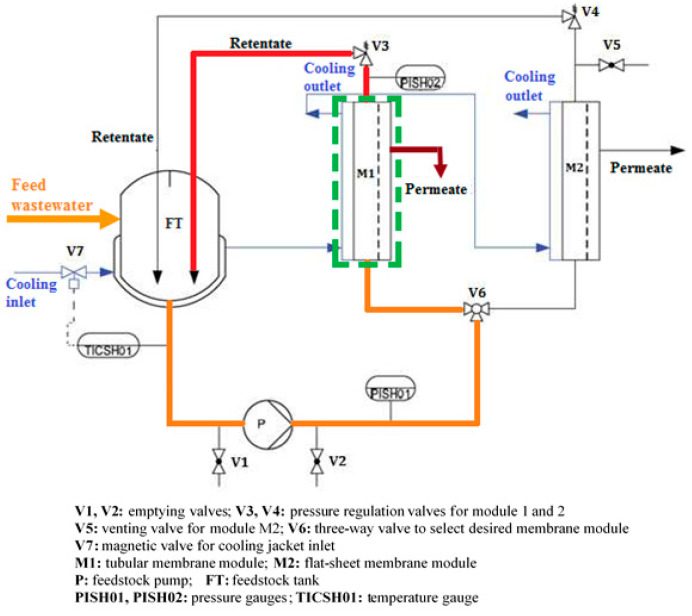
Flow scheme of the bench-scale NF membrane plant.

Concentration–purification experiments of quinoa extracts were conducted in a bench-scale membrane system (scheme described in Figure 3). For this goal, a proper flat-sheet NF membrane was selected and inserted in a plate and frame module (Prozesstechnik GmbH, Basel, Switzerland).

The characteristics of the chosen membrane are given in Table 1. Additionally, a polymeric network spacer (2 mm mesh diameter) was arranged in the plate and frame module to promote adequate turbulence over the membrane surface to minimize concentration polarization [29].

The bench-scale membrane plant was provided with an overpressure and overtemperature protection setup. To avoid corrosion, metal elements in direct contact with the feedstream—membrane module, tank, pipes, and accessories—were fabricated using 316 L stainless steel, whereas concentrate and permeate pipes were made of chemical-resistant polyethylene. The system was equipped with a double-walled tank (5 L) and modules, connected to a cooling circuit (chiller PolyScience Model 7306) to maintain the desired temperature (±0.1 °C) via an electronic PID controller (UT100, Yokogawa, Tokyo, Japan). The operating pressure could be set accurately (PTM set point ± 0.01 bar) with a poppet valve (SS-R4512MM-SP, Swagelok, Solon, OH, USA) and displayed by a digital manometer (Ceraphant T PTC31, Endress+Hauser, Reinach, Switzerland). The diaphragm pump (Hydra-Cell model D-03) flow rate was accurately established with a flow valve (±0.1 L/h).

Beforehand, the membrane was preconditioned in situ with Milli-Q^®^ water until the pure water flux remained stable. The membrane’s initial permeability (m_w_, see Table 1) was determined by measuring the pure water flux of the membrane within its admissible pressure range, keeping the temperature constant (25 ± 0.5 °C), and setting the flow rate to the minimum allowed by the pump (1.2 L/min).

Subsequently, the quinoa extract sample (400 mL)—after extraction, centrifugation, and filtration—was added to the feed tank. NF experiments were conducted in semicontinuous mode: the concentrate stream was continuously returned to the feed vessel, while the permeate was collected in a probe. At the end of every test, samples of both streams (permeate and concentrate) were taken for immediate analysis or stored for later examination.

The membrane rejection towards saponins and phenolic compounds was thus calculated using the following expression:

(1) $$R_{i}\left(\%\right)=\left(1-\frac{C_{p,i}}{C_{f,i}}\right)\times 100$$

where *C*_*p*,*i*_ is the concentration of compound I in the permeate stream and *C_f,i_* represents the concentration of solute I in the feed stream.

The effects of the key operating parameters of the purification process—operating pressure and tangential flow velocity—on the NF membrane performance, particularly on the permeate flux (Jp, L/hm^2^) and membrane rejection efficiency (*R_i_*, %), were examined. Combinations of the different operating conditions (P: operating pressure, v_t_: tangential velocity) set during the NF purification of quinoa hull extracts are reported in Table 2.

After completion of each experiment, the NF membrane was subjected to a cleaning-in-place procedure, adapted from methods from previous studies by the authors [43]. In summary, this procedure involved first three rinses with Milli-Q^®^ water at 25 °C at the minimum operating pressure (2.5–2.8 bar) and minimum feed flow rate (1.2 L/min) for 5 min to remove any organic and/or colloidal matter superficially deposited on the membrane surface, as well as on the feed spacer. This was followed by a chemical cleaning step with 1 L of 0.05% *w*/*w* NaOH plus 0.05% *w*/*w* sodium dodecyl sulfate (SDS) solution, recirculating it through the equipment for 15 min at 25 °C, at the minimum operating pressure (2.5–2.8 bar) to avoid causing further fouling due to obstruction of membrane pores, and the minimum feed flow rate (1.2 L/min), concluding with a final triple rinse for 5 min to remove any remaining cleaning reagent.

### 2.5. NF Purification Process Modelling and Optimization

The performance of the chosen NF membrane for separation, concentration, and purification of high-added-value compounds—saponins and phenolic compounds—from quinoa hull extracts was further modeled and optimized. For this goal, a 3-level factorial model was established for the design of experiments (DE); that is, 3 × 2 levels including the central point. This involved 3 response variables, which required the performance of 11 experimental runs with 2 repetitions of the central point to be able to confirm the reproducibility of the method. Response variables comprised saponins rejection (Rsap%), total phenolic compounds rejection (RTPhs%), and the permeate flux yielded by the membrane (Jp(t)).

Modeling and optimization were performed using Statgraphics Centurion XVI.I statistical software. Several considerations related to the operating conditions of the membrane in terms of pressure and tangential velocity were taken into account to establish the minimum and maximum values for the input factors to be studied. The range of the main operating variables of the selected NF membrane is given in Table 3.

The tangential flow range studied was considered to be high enough to cause turbulent flow at the inlet of the feedstream into the membrane module, measured by the Reynolds number (N_Re_).

N_Re_ was calculated with the equation by Mott [29,44] for flow in rectangular sections:

(2) N_Re_ = υ(4R) ρ/μ

where R corresponds to the hydraulic radius of the module, calculated as A/P, in which A is the area (0.02 m^2^) and P is the perimeter (0.68 m); v is the average speed of the fluid flow through the surface (that is, the tangential velocity v_t_), ρ is the fluid density (kg/m^3^), and μ is the fluid viscosity (kg/m s). The resulting v_t_ for the different volumetric flow values tested (3 × 10^−5^, 5 × 10^−5^, and 7 × 10^−5^ m^3^/s) were equal to 15, 25, and 35 m/s, which correspond to N_Re_ equal to 1.74 × 10^6^, 2.90 × 10^6^, and 4.06 × 10^6^. The flow regime promoted over the membrane was in turbulent range, given the N_Re_ obtained.

This strategy permitted us to gather sufficient data from the DE to determine the optimal performance of the chosen NF membrane in terms of flux production and target compound separation. The response surface methodology (RSM) was used to interpret the obtained results and evaluate the relative impacts of the main operating factors on the membrane system performance [44].

The NF performance response was described by a second-order polynomial equation. This permitted rendering the response surface (RS) for each studied response variable accurately, with concavities and/or convexities. This mathematical model comprises several coefficients (*b_i_*) that could be inferred from the experimental data fitting. The RS presents the following form:

(3) $$y=b_{0}+\sum b_{i}x_{i}+{\sum b}_{ii}x_{i}^{2}+{\sum b}_{ij}x_{i}x_{j}$$

where *y* is the response function and *x_i_* are the input factors involved and evaluated.

The optimum value for each *x_i_* factor could be determined by differentiating Equation (3):

(4) $$\frac{\delta y}{\delta x_{i}}=b_{i}+2b_{ii}x_{i}+b_{ij}x_{j}=0$$

## 3. Results

### 3.1. Extraction of Saponins and Phenolic Compounds from Milled Quinoa Hulls

The results obtained from extraction experiments of the target added-value compounds—saponins and phenolic compounds—are reported in Table 4, where the extraction yields of both target compounds are given. Extraction tests were carried out with a 60 wt% ethanol–water mixture and a 1:10 solid–liquid ratio for 24 vs. 48 h, using magnetic or orbital agitation. The temperature was fixed at 40 °C, and the stirring speed (200 rpm) ensured a complete homogeneous mix in the reactor.

**Figure 4:**
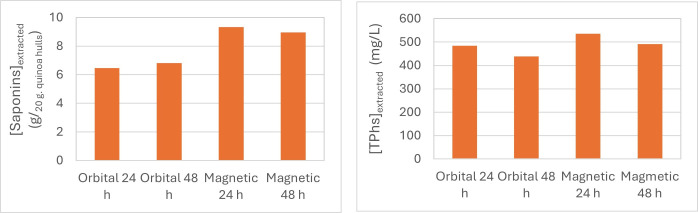
Added-value compound extraction using orbital vs. magnetic agitation at 40 °C, 200 rpm stirring speed, 60 wt% ethanol–water mixture, and 1:10 solid–liquid ratio. Left: saponin concentration; right: total phenolic compound (TPh) concentration.

As can be noted, the extraction of both saponins and phenolic compounds from quinoa subproduct (hulls) was maximized upon magnetic agitation during a maximum of 24 h. In detail, regarding saponin extraction, magnetic stirring ensured better results if compared with orbital agitation, that is, 53.4–55.2 g/L in contrast to 45.2–48.7 g/L, which represented an up to 22.1% concentration increment (see Figure 4). Moreover, an increase in extraction time beyond 24 h resulted in a yield of 9.33 g/20 g. quinoa (466.6 mg/g quinoa) but did not provide significantly better results regarding the type of agitation. Increasing contact time did not result in major saponin extraction in the case of magnetic stirring, whereas in the case of orbital shaking, it only resulted in a 7.7% extraction increase of these compounds, which would not be significant from a technical–economic point of view.

In contrast with the attained results, Tan et al. achieved 65% extraction with a 70% water/ethanol solvent mixture using a 20:1 solvent-to-solid ratio. In contrast, saponins extracted by Gil-Ramírez et al. [45] with pressurized hot water extraction (PHWE) at 195 °C achieved 15.4 mg/g raw material. Taco et al. [10] obtained 110–170 mg/g saponins from quinoa husks of *Tunkahuan* and *Pata de Venado* varieties by using deep eutectic solvents or 80% ethanol/water, whereas Norouzpour et al. [46] attained 78 mg/g with methanol at a 1:5 S–L ratio. Tan et al. [47] otherwise attained a 22% extraction yield of saponins from *Camellia oleifera Abel* using deep eutectic solvent (DES) comprising L-proline, glycerol, and sucrose at a 4:10:1 molar ratio, diluted with water at 7:3 (*w*/*w*), and 15% to 18% extraction yields with DES comprising choline chloride, betaine, L-proline, glycerol, and sucrose.

On the other hand, in the case of the extraction of total phenolic compounds (TPhs) from milled quinoa hulls, both types of agitation offered similar behavior, though magnetic stirring provided better results in contrast to orbital agitation (Table 4). After 24 h extraction with magnetic agitation, 536 mg/L TPhs were extracted vs. 484 mg TPhs/L in the case of orbital mixing, that is, a 10.7% increment (see Figure 4). An increase in extraction time beyond 24 h did not yield better results for either type of mixing; it did not result in an increase in TPh extraction in the case of magnetic stirring, and similar results occurred after 48 h of orbital mixing, not improving the amount of TPh compound extracted (see Figure 4).

### 3.2. Concentration and Purification of Target Compounds NF Membrane Response Analysis

Firstly, the pure water permeability of the membrane (m_w_) was determined by measuring the flux at 25 °C and 3 L/min over the range of operating pressures (1–16 bar) [44]. A value for mw equal to 8.0 ± 0.5 L/h∙m^2^ bar was attained (Table 1). This value served as a reference for checking the recovery of the membrane performance after each experimental test (m_w_’) and the applied cleaning procedure.

After this, NF experiments with quinoa hull extracts were performed. The purpose of the experimental design (DE) was to analyze, model, and optimize the performance of the NF membrane selected for the concentration and purification of saponins and phenolic compounds present in the previously obtained quinoa hull extract. This goal focused on the key operating variables, comprising the operating pressure and tangential velocity over the membrane. Rejection of saponins (Rsap, %), total phenolic compounds (RTphs, %), and the membrane permeate flux value (Jp, L/hm^2^) yielded by the NF membrane were registered as key response variables. NF operation experiments were performed, taking into account the factors and levels indicated in Table 2 and Table 3, with triplication of the central point conditions performed to check the reproducibility of the DE. Concentration percentages of saponins ([sap_permeate_], %) and phenolic compounds ([TPhs_permeate_], %) obtained in the permeate, and the resulting rejection performance of the selected NF membrane, as well as the permeate flux and membrane fouling index, are reported in Table 5.

Moreover, the volume concentration factor (VCF)—the ratio of final concentrate stream volume to initial feed sample volume—was calculated as:

(5) VCF = Vinitial/Vconcentrate

A VCF of up to 4 was achieved during the experiments, as 400 mL of centrifuged and pre-filtered milled quinoa extract samples were initially added to the feed tank, and experiments were run until at least 300 mL of permeate flux was obtained.

In terms of the rejection performance of the selected NF membrane, in all tests, it was observed that, as a common pattern, the percentage of saponins could be concentrated in a major proportion in the permeate, with the highest value of up to 78.0% achieved upon P = 4 bar and v_t_ = 25 m/s. Also, a 70.1% saponin concentration in the permeate stream was attained upon P = 16 bar and v_t_ = 15 m/s, but it should be underlined that higher pressure was applied. On the contrary, the lowest percentage of saponins concentrated in the permeate, at 43.3%, was obtained at the minimum operating conditions examined, that is, P = 4 bar and v_t_ = 15 m/s, indicating that fouling developed over the NF membrane could be negatively affecting its rejection performance.

Regarding the concentration of total phenolic compounds, a similar trend to that for saponins was observed since, in all tests, the highest concentration was obtained in the permeate, upon P = 10 bar and v_t_ = 15 m/s, up to a value of 73.5%, followed by conditions at P = 16 bar and v_t_ = 35 m/s with 66.9%. Again, in the latter case, minor concentration was measured in the permeate at a higher pressure, pinpointing fouling developing on the membrane surface acting as a double barrier against the passage of these compounds [29]. On the contrary, the lowest percentages, 45.8% and 49.6%, respectively, were yielded upon P = 4 bar and v_t_ = 15 m/s and P = 10 bar and v_t_ = 35 m/s.

**Figure 5:**
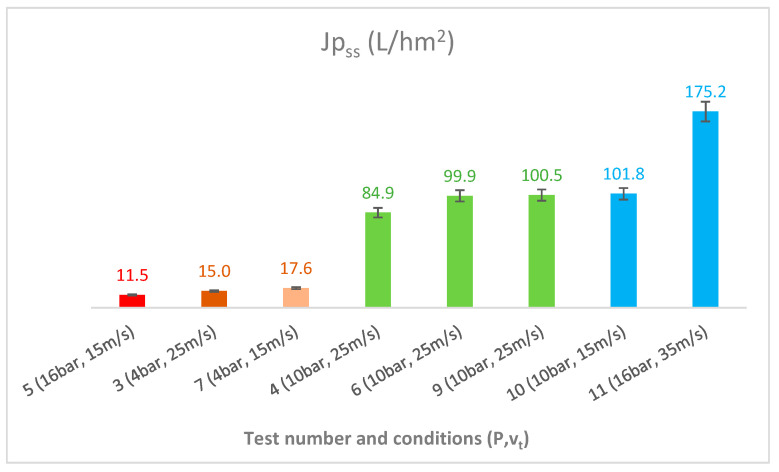
Permeate flux values yielded in steady-state (Jp_ss_, L/hm^2^) upon variation of key NF membrane operating variables: operating pressure (P, bar) and tangential velocity over the membrane (v_t_, m/s).

**Figure 6:**
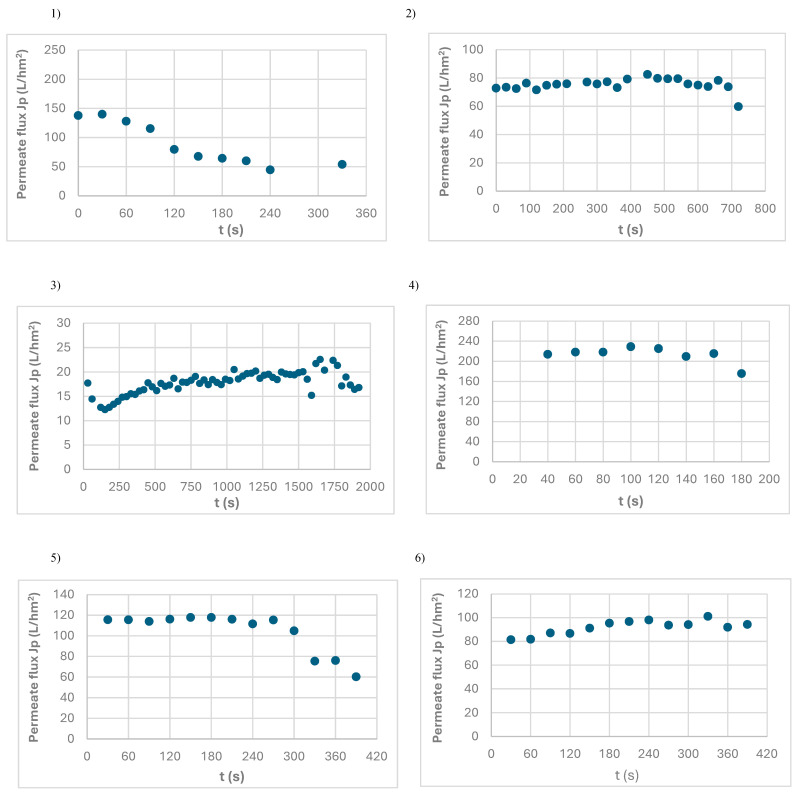

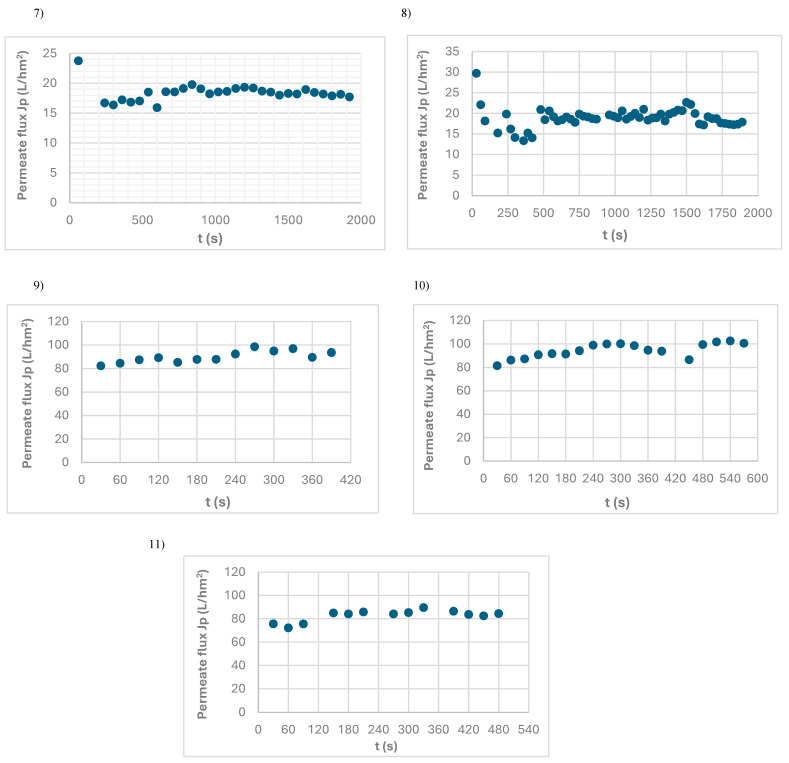
Dynamic NF flux patterns for purification experiments of quinoa hull extracts.

On the other hand, the results of the NF membrane permeate flux value (L/hm^2^) are summarized in Figure 5. Flux maximization plus stability is of utmost importance for the process’s cost efficiency and thus was the goal of this research work [43,44]. Dynamic NF flux patterns for each experiment are given in Figure 6.

As can be seen in Figure 5 and Figure 6, high and stable permeate flux could be attained at steady state (Jp_ss_) by running the NF process at P = 10 bar and v_t_ = 25 m/s (tests 4, 6, and 9), which permitted us to achieve a value of Jp = 84.9–100.5 L/hm^2^, as well as at P = 10 bar and v_t_ = 15 m/s (test 10), which also ensured high and stable permeate flux, reaching a plateau of Jp of up to 101.8 L/hm^2^. These conditions ensured that a steady flux could be yielded and, therefore, stable and safe operating conditions would be maintained in terms of membrane performance. On the other hand, test 10 (P = 10 bar, v_t_ = 15 m/s) presented a lower percentage of permeate flux drop (−23.4%), which implied an increase in the initial flux due to swelling of the membrane polymer upon contact with the fluid [29].

As can be observed in Figure 6, concentration polarization, shown by a rapid initial flux decline, was noted (experiments 7 and 8), but then flux stabilized as no fouling developed over the membrane at low-pressure conditions, which indicates low convective force on the active layer.

Furthermore, by increasing the operating pressure up to P = 16 bar and simultaneously increasing the v_t_ over the NF membrane to 35 m/s, a high NF membrane flux of up to 175.4 L/hm^2^ and steady-state stability could be attained. This was the result of the sufficient sweeping effect of the higher tangential flow set over the membrane that compensated and equilibrated the higher convective force at increased pressure [29]. Likewise, the conditions yielding the lowest fouling were observed at v_t_ = 25 m/s and P = 4–10 bar, with 0.4 and 0.90 (h^−1^) fouling build up, respectively, for which fouling was found to also behave reversibly. This is quite critical for the safe and stable scale-up of the proposed membrane process, as membrane fouling is the most important limiting factor for the application of membrane technology for purification treatments [29]. Fouling leads to a reduction in productivity over time and causes significant shortening of the lifetime of membrane modules; therefore, fouling can make the process unsustainable [29]. Moreover, fouling also alters the membrane selectivity and thus its rejection efficiency.

On the contrary, high pressure but insufficient tangential velocity on the membrane—P = 16 bar, v_t_ = 25 m/s and P = 16 bar, v_t_ = 15 m/s—resulted in the highest permeate flux loss at 60.8% and 47.9% respectively, as a result of the highest fouling rates per hour, with 28.62 and 21.50 (h^−1^) fouling index b, respectively. In these cases, some semi-irreversible fouling was also measured, negatively affecting the process performance, pinpointing the challenge when adopting these operating conditions.

### 3.3. Process Modeling and Optimization for NF Membrane Purification of Saponins and Phenolic Compounds

Prediction and control of membrane fouling is critical for effective design, successful scale-up, steady operation, and optimization of the proposed membrane concentration + purification of saponins and phenolic compounds from quinoa hull extracts. This task is usually difficult and complex. Multifactorial analysis of the response function—permeate flux (Jpss)—dependent on the key input factors of the NF process was carried out. This statistical analysis allowed us to quantitatively determine the different potential complex conjugate impacts on the NF membrane operation, thus estimating their significance and effects for control and optimization.

**Figure 7:**
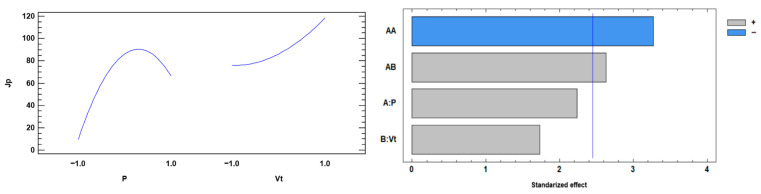
Effects of main operating variables (P, v_t_) on the steady-state permeate flux Jpss (L/hm^2^) of the NF membrane for saponins and TPhs extract concentration + purification (**left**), and Pareto chart for standardized impacts (**right**).

For this purpose, a three-level factorial model comprising the relevant input factors on the NF membrane performance—P and v_t_—was completed. Analysis of variance (ANOVA) of the data gathered in the experiments was subsequently done, and response surface methodology (RSM) was finally implemented to model and optimize the NF process. The impacts of the operating factors—operating pressure (P) and tangential velocity (v_t_)—on the permeate flux of the NF membrane (Jp, L/hm^2^) were statistically examined, establishing a central point (0) and two limit levels (minimum and maximum, −1 and +1) for each operating factor, as previously indicated in Table 3. The results obtained are presented in Figure 7.

As can be observed in Figure 7, the variation of the operating pressure and its impact on the permeate flux yielded by the NF membrane reached a maximum when the pressure value slightly exceeded the central point (≈0.1), that is, approximately at P equal to 11 bar. At this pressure value, a critical permeate flux was reached, which indicated that if the pressure was increased above this value, a greater dynamic accumulation of fouling would occur on the membrane, thus increasing fouling and decreasing the boundary flux obtained (Jpss) [29]. In addition to this, the increase in tangential velocity had a direct and proportional impact on the permeate flux since, by increasing it to a certain value, a higher steady-state permeate flux was steadily attained. This is due to the increase in the drag force caused by enhanced turbulence associated with the increase in v_t_. Consequently, the accumulation of particles in the membrane could be minimized, thus reducing fouling [29].

The Pareto chart in Figure 7 shows the response variables with a positive or negative quantified impact on the permeate flux of the membrane: the vertical line divides the variables that are not significant (to the left of the line) and those that are significant (beyond the line). As can be noted, operating pressure P (squared) had a relevant significance in the permeate flux yielded by the selected NF membrane (*p*-value of 0.0301), as well as the tangential velocity v_t_ interaction with pressure (*p*-value of 0.0389). Thus, it was proven relevant to consider these effects, whereas the rest of the input factors were not found to have a significant effect on Jpss and therefore were not taken into account for the model.

**Figure 8:**
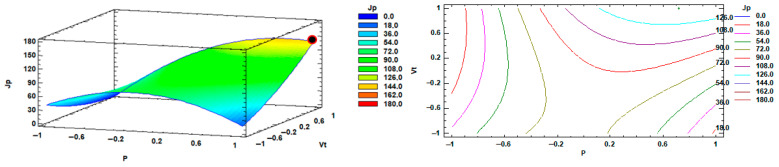
Response surface (**left**) and contour plot (**right**): effect of the operating variables—P and v_t_—on Jp (L/hm^2^) yield.

Within the gathered information, response surface (RS) and contour plots of the effect of the operating variables—P and v_t_—on NF flux Jp yield (L/hm^2^) were obtained (Figure 8). For the proposed NF concentration + purification of saponins and phenolic compounds from quinoa hull extracts, this gave relevant information on quinoa hulls’ extract interactions with the chosen membrane regarding membrane fouling, with the goal of successfully implementing the process at an industrial scale [29]. Subsequently, optimized values of the input factors affecting the performance of NF concentration + purification of saponins and phenolic compounds from quinoa hull extracts, in terms of flux obtained (Jpss), were also obtained. Results of NF process optimization are reported in Table 6.

Optimization of the process permitted maximizing the permeate flux of the NF membrane up to 175.4 L/hm^2^, upon the optimal values of the input factors, that is, an operating pressure not exceeding 12.9 bar plus a tangential velocity of 25 m/s over the membrane (Table 6). This is illustrated in the response surface and contour plots given in Figure 8. The attained optimized results are of paramount relevance since maximum membrane productivity and stable permeate flux during operation could be ensured, pinpointing that fouling on the NF membrane could be successfully minimized and controlled, thus positively impacting the lifetime of the membrane, as well as the performance of the operation. In this way, the times and frequencies of stops in the process to carry out the cleaning of the membrane would be minimized, reducing energy and reagent consumption, which directly impacts the costs of the operation. Complete membrane permeability restoration could be achieved after the applied cleaning protocol in these conditions [43,44]. Optimized values of the input factors affecting saponins and phenolic compounds from quinoa hull extracts’ NF purification performance (Jpss) were validated by the proposed methodology. This allowed fouling build-up minimization and thereby the maximization of the Jpss up to almost ten-fold its value, from 16.4–17.7 L/hm^2^ up to 175.4 L/hm^2^. It was therefore shown that proper condition optimization of this highly polar and hydrophilic TFC polyamide active layer membrane could trigger the yielded flux performance [44].

The ANOVA of the obtained model for NF membrane concentration + purification of saponins and TPhs from quinoa hull extracts showed a *p*-value of 0.01, thus below 0.05, which confirms a significant dependence of NF steady-state flux (Jpss) on squared operating P, as well as v_t_ and P parameter interactions, with 95.0% confidence. The R^2^ statistics indicated that the model accounts for 81.4% of Jpss variation with P and v_t_ (Figure 8). In detail, the mean absolute error (MAE) was 1.12, and the standard residue deviation was calculated as 1.86. Moreover, the Durbin–Watson statistic pinpointed random variation of the residuals, given that its value was close to 2 (1.743), with the *p*-value equal to 0.11, ensuring no serial autocorrelation among residuals with 95.0% confidence. To sum up, negligible Lag 1 residuals autocorrelation (0.099), tending toward zero, confirmed no significant structure uncorrelated by the inferred model. The values attained for these statistical parameters proved the validity of the proposed model for NF Jpss as a function of P and v_t_.

**Figure 9:**
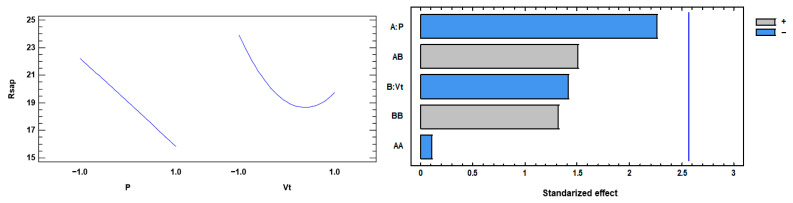
Effects of main operating variables (P, v_t_) on saponin rejection (%Rsap) by the NF membrane (**left**), and Pareto chart for standardized impacts (**right**).

In addition to this, the impacts of the main operating variables (P, v_t_) on the rejection of the target added-value compounds of interest were analyzed. The results of separation yield toward saponins (%Rsap) by the selected NF membrane of the previously obtained extract are shown in Figure 9. The Pareto chart for the standardized impact of the input factors on %Rsap is also reported. An inversely proportional impact trend of pressure on rejection of saponins (Rsap) was observed; that is, a higher concentration of saponins was attained in the retentate stream at low pressure values (left chart in Figure 9). Consequently, as the operating pressure was augmented, saponins were displaced in higher concentrations to the permeate side of the NF membrane. Regarding the tangential velocity, a decrescent linear trend of Rsap with v_t_ was observed for the range within low tangential velocity values (15 to 25 m/s); that is, the rejection of saponins decreased. Then, as v_t_ over the membrane increased, an inflexion point was reached on the rejection of saponins, such that for v_t_ above 25 m/s, the tendency of saponin rejection increased. A guided focus on these results suggests that the driven secondary resistance layer constituted by fouling and concentration polarization was eliminated due to sufficient effective tangential sweeping of these compounds over the NF membrane surface.

Moreover, a guided focus on Figure 9 allowed us to confirm operating pressure as the input factor that most significantly influenced the percentage of saponin rejection.

**Figure 10:**
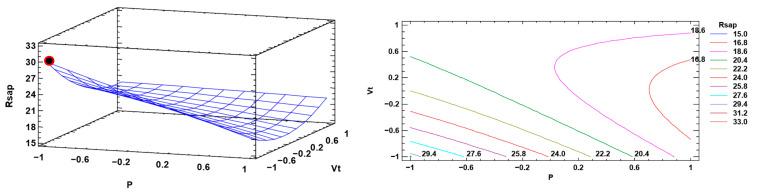
Response surface (**left** graph) and contour plot (**right** graph): effect of the operating variables—P and v_t_—on saponin rejection (%Rsap) yield.

Subsequently, response surfaces (RS) and contour plots of P vs. v_t_ impacts on NF purification of saponins from quinoa hull extracts in terms of saponin rejection (%Rsap) are hereafter reported (Figure 10). The impacts of the operating factors, that is, pressure (P) and tangential velocity (v_t_), on the rejection of saponins are shown, establishing a central point and the limit levels for each previously determined operating factor.

Data optimization allowed us to predict a value to maximize the rejection of saponins, estimated with statistical software, equal to 29.8%, meaning the concentration of up to 70.2% of saponin compounds in the permeate stream (38.6 g/L), by setting the response variables at the optimal values of 4 bar and 15 m/s for operating pressure (P) and tangential velocity (v_t_), respectively. This is summarized in Table 7. On the other hand, a strategy based on the maximum operating P and central point for v_t_ would lead to minimum %Rsap, which would allow the concentration of saponins in the permeate stream to reach 84.5%. In the research study by Hilares et al. [48], the authors proposed hydrodynamic cavitation followed by MF using tubular ceramic (Al_2_O_3_) membranes (1.1 µm pore size), resulting in 74% saponin recovery in the permeate. Thereafter, the permeate was passed through UF (3 nm, ZrO_2_ membrane), which recovered 32% of saponin in the permeate, and subsequently by NF (600–800 Da, PA-TFC), 92% of saponin was recovered in the retentate, which was then subjected to spray-drying, producing a powder with a 54% saponin content. However, these authors did not report nor optimize the dynamic fouling and membrane flux, which are key for process scale-up.

**Figure 11:**
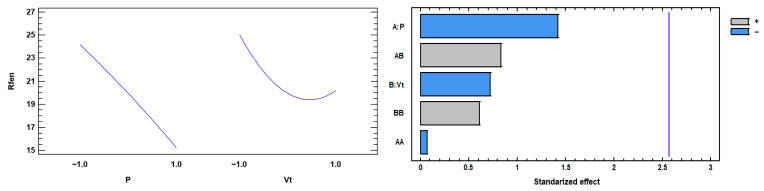
Effects of main operating variables (P, v_t_) on phenolic compound rejection (%RTPhs) by the NF membrane (**left** chart), and Pareto chart for standardized impacts (**right** chart).

**Figure 12:**
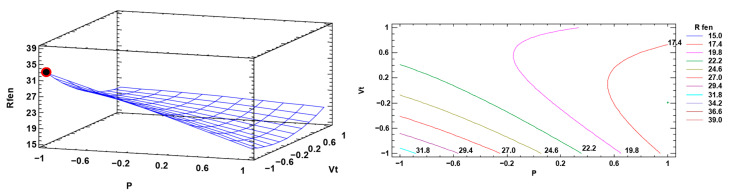
Response surface (**left**) and contour plot (**right**): effect of the operating variables—P and v_t_—on phenolic compound rejection yield (%RTPhs).

Moreover, the effects of the main operating variables (P, v_t_) on the rejection of phenolic compounds (%RTPhs) by the selected NF membrane were analyzed and optimized. The results are reported in Figure 11. The standardized Pareto charts for the quantified impacts of the key operating variables (P, v_t_) attained on NF membrane RTPhs of the obtained extract are also given. Response surface and contour plots for the effects of the input factors—P and v_t_—on the rejection yield of phenolic compounds (%RTPhs) are additionally given in Figure 12.

In the Pareto chart (Figure 11), it is shown that the rejection of total phenolic compounds was found to be more significantly affected by the operating pressure of the membrane (*p*-value = 0.2215), with a lower impact of tangential velocity (*p*-value = 0.4804), similarly to what was observed for both main operating variables (P, v_t_) in the case of saponin rejection. Regarding the rejection of phenolic compounds, similar trends were observed regarding both operating variables P and v_t_, to those recorded for the rejection of saponins (Figure 11 and Figure 12). The operating pressure was found to have a negative effect on the rejection of phenolic compounds, as a major concentration passed through the NF membrane at higher pressure conditions. Otherwise, the tangential velocity over the membrane surface also analogously impacted NF performance for saponins, exhibiting a range of low tangential velocity below 25 m/s, for which a negative linear impact trend on RTPhs was registered, but above which RTPhs rejection increased as a result of fouling and concentration polarization over the membrane surface being swept by effective tangential force [29,44].

To sum up, the optimal value predicted to minimize the rejection of phenolic compounds was found to be 15.2% when the response variables were 14 bar operating P and 23.2 m/s v_t_ (Table 8). This permitted up to 84.3% of phenolic compounds to be concentrated in the obtained permeate stream.

The modeled and optimized results highlight that the proposed purification scheme could be successfully run under mild pressure conditions, as well as tangential velocity over the membrane, which would allow counterbalancing of the economic return of the NF process. Moreover, the results of the optimization and modeling of the present research work could be extrapolated based on the correlations obtained, since the physico-chemical composition of the raw quinoa material may be quantitatively different depending on the different regions, but qualitatively similar. The obtained results are very relevant for the potential scale-up of the proposed concentration and purification process of saponins and phenolic compounds from quinoa hull by-products.

## 4. Conclusions

In this work, the obtention of saponin- and phenolic compound-rich extracts from a by-product of quinoa agro-industry—quinoa hulls—via green solvent extraction and NF purification was investigated, modeled, and optimized. It is key for quinoa cultivation and its widespread expansion in the global market to comply with the circular economy scheme to become a green agro-food industry.

The effects and impacts of the main operating variables (P, v_t_) on rejection performance of the selected NF membrane toward saponins and phenolic compounds of the previously obtained extract with green solvent were successfully optimized and modeled using statistical multifactorial analysis and response surface methodology.

Optimization of both operating pressure (P) and tangential velocity (v_t_) at 4 bar and 15 m/s permitted the recovery of up to 84.5% saponin compounds in the permeate stream, obtaining a purified stream enriched in 38.6 g saponins/L. On the other hand, 84.3% phenolic compounds could also be recovered in the permeate, reaching up to 767.4 mg TPhs/L.

Moreover, the dynamic performance of the chosen NF membrane during purification was optimized and modeled by response surface methodology. This allowed fouling build-up minimization and thereby the maximization of the membrane flux productivity almost tenfold, up to a stable value as high as 175.4 L/hm^2^. This is of paramount importance and would allow us to operate the NF membrane purification process at stable steady-state conditions, which ensures the full recovery of the membrane performance in terms of rejection yield and permeability. These results are key for the potential technical and economic viability of the proposed extraction and purification scheme to obtain high-added-value bioactive standardized purified extracts from quinoa by-products on an industrial scale, which might encounter future markets in the pharma, food, cosmetic, and biotechnology sectors.

## Figures and Tables

**Table 1:** Physico-chemical characteristics of the selected NF membrane.

Parameter	Value
Supplier	GE Water & Process Tech.
Model	NF (DK)
Active surface (cm^2^)	200
Permeability (m_w_) (L h^−1^ m^−2^ bar^−1^)	8.0 ± 0.5
Configuration	Flat-sheet
Chemical structure	Thin-film composite (TFC)
Chemical composition	Polyamide/polysulfone
Surface nature	hydrophilic
MWCO * (Da)	300
Mean pore diameter (D_p_), nm	0.5
Max. Pressure (bar)	40
Max. Temperature (°C)	50
pH range	1–11
Module spacer	Flat, 45 mil paralell

* MWCO: molecular weight cut-off.

**Table 2:** Combinations of different operating conditions (P: operating pressure, v_t_: tangential velocity) during NF separation, concentration, and purification of quinoa hull extract.

Test	*P* (Bar)	*v_t_* (m/s)
1	16	25
2	10	35
3	4	25
4	10	25
5	16	15
6	10	25
7	4	15
8	4	35
9	10	25
10	10	15
11	16	35

**Table 3:** Input factors and ranges for NF separation, concentration, and purification of saponins and total phenolic compounds from quinoa hull extracts.

Input Factor	Minimum (−1)	Central (0)	Maximum (+1)
*P* (bar)	4	10	16
*v_t_* (m/s)	15	25	35

**Table 4:** Results from extraction experiments of saponins and phenolic compounds from milled quinoa hulls using different agitation types and extraction time.

Stirring Type	Extraction t (h)	[Saponins], g/L	g_saponins_/20 g _quinoa hulls_*	[TPhs], g/L	g_TPhs_/20 g _quinoa hulls_*
Orbital	24	45.2	6.46	0.484	0.076
	48	48.7	6.82	0.439	0.061
Magnetic	24	55.2	9.33	0.536	0.091
	48	53.4	8.97	0.491	0.082

* 20 g quinoa hulls (1:10 *w*/*v* ratio).

**Table 5:** Results of NF performance in the purification of quinoa hull extracts: permeate flux, membrane fouling index, and rejection efficiencies.

Test	*Jp*_o_ (L/hm^2^)	*Jp*_ss_ (L/hm^2^)	*Fouling index b*, (h^−1^)	*R*_sap_ (%)	*R*_TPhs_ (%)	[*sap*_permeate_] (%)	[*TPhs*_permeate_] (%)
1	137.8	44.7	28.6	15.5	15.3	65.2	56.7
2	72.7	59.7	1.9	21.3	16.5	63.3	49.6
3	17.7	16.4	0.9	20.8	17.4	78.0	61.6
4	78.7	84.4	0.4	17.8	16.6	68.8	59.3
5	115.7	60.2	21.5	19.2	16.8	70.1	56.2
6	84.5	89.6	14.0	16.6	16.3	64.4	59.2
7	23.8	17.7	19.9	31.7	35.9	43.3	45.8
8	29.7	17.4	18.7	19.5	24.4	52.3	60.0
9	81.8	91.9	13.9	24.5	33.7	46.9	56.6
10	86.2	100.4	14.2	20.7	21.9	56.3	73.5
11	214.1	175.4	5.3	18.2	19.0	61.7	66.9

Rejection of saponins (Rsap, %), rejection of total phenolic compounds (RTphs, %), membrane permeate flux (initial Jp,o vs. steady-state Jp,ss L/hm^2^), percentages of saponins [sappermeate] (%) and phenolic compounds [%TPhs permeate] (%) in the permeate.

**Table 6:** Optimized response variables’ values for optimal permeate flux of NF concentration and purification of saponins and total phenolic compounds from quinoa hull extracts.

Variable	Point	Value	*J_p_*_optimized_ (L/hm^2^)
*P* (bar)	0.718	12.9	175.4
*v_t_* (m/s)	1	25.0	

**Table 7:** Optimal response variables’ values for NF concentration and purification of saponins from quinoa hull extracts.

Variable	Point	Value	*%*sap _permeate_ (%)
*P* (bar)	−1	4.0	70.2
*v_t_* (m/s)	−1	15.0	

**Table 8:** Optimal response variable values for NF concentration and purification of phenolic compounds from quinoa hull extracts.

Variable	Point	Value	*TPhs*_permeate_ (%)
*P* (bar)	1	14.0	84.3
*v_t_* (m/s)	−0.188	23.2	

## Data Availability

The original contributions presented in this study are included in the article. Further inquiries can be directed to the corresponding author.
